# miRNA Regulation of Gene Expression: A Predictive Bioinformatics Analysis in the Postnatally Developing Monkey Hippocampus

**DOI:** 10.1371/journal.pone.0043435

**Published:** 2012-08-28

**Authors:** Grégoire Favre, Pamela Banta Lavenex, Pierre Lavenex

**Affiliations:** Laboratory of Brain and Cognitive Development, Department of Medicine, University of Fribourg, Fribourg, Switzerland; The Centre for Research and Technology, Hellas, Greece

## Abstract

Regulation of gene expression in the postnatally developing hippocampus might contribute to the emergence of selective memory function. However, the mechanisms that underlie the co-regulation of expression of hundreds of genes in different cell types at specific ages in distinct hippocampal regions have yet to be elucidated. By performing genome-wide microarray analyses of gene expression in distinct regions of the monkey hippocampal formation during early postnatal development, we identified one particular group of genes exhibiting a down-regulation of expression, between birth and six months of age in CA1 and after one year of age in CA3, to reach expression levels observed at 6–12 years of age. Bioinformatics analyses using NCBI, miRBase, TargetScan, microRNA.org and Affymetrix tools identified a number of miRNAs capable of regulating the expression of these genes simultaneously in different cell types, i.e., in neurons, astrocytes and oligodendrocytes. Interestingly, sixty-five percent of these miRNAs are conserved across species, from rodents to humans; whereas thirty-five percent are specific to primates, including humans. In addition, we found that some genes exhibiting greater down-regulation of their expression were the predicted targets of a greater number of these miRNAs. In sum, miRNAs may play a fundamental role in the co-regulation of gene expression in different cell types. This mechanism is partially conserved across species, and may thus contribute to the similarity of basic hippocampal characteristics across mammals. This mechanism also exhibits a phylogenetic diversity that may contribute to more subtle species differences in hippocampal structure and function observed at the cellular level.

## Introduction

The hippocampal formation is comprised of a group of cortical regions located in the medial temporal lobe, which is essential for spatial and episodic memory functions [1], [2], [3], [4], [5]. Recent data suggest that the gradual emergence of different types of “hippocampus-dependent” memory processes may be dependent on the differential maturation of distinct hippocampal circuits during early postnatal life [6], and that major changes in gene expression [7] are concomitant with structural changes occurring at different ages in distinct hippocampal regions [6], [8]. Using stringent analysis criteria, we previously identified a number of genes exhibiting differences in expression between distinct hippocampal regions across developmental ages [7]. In the current study, we confirmed the existence of a large group of genes whose expression decreased between birth and six months of age in CA1, and after one year of age in CA3, to reach expression levels observed in 6–12-year-old monkeys. In addition to the previously identified group of genes expressed in astrocytes [7], we found a number of genes expressed in neurons and oligodendrocytes that exhibited similar patterns of expression. We used several bioinformatics tools, including NCBI (National Center for Biotechnology Information), miRBase, TargetScan, microRNA.org and Affymetrix bioinformatics software, to perform predictive analyses and determine possible mechanisms of co-regulation of the expression of hundreds of genes expressed in different cell types. Our analyses specifically considered the involvement of miRNAs (small RNA molecules acting as post-transcriptional regulatory elements), which have been shown to play a major role in developmental processes [9], [10]. Indeed, previous studies revealed the prominent role of miRNAs in the early stages of neuronal fate determination and differentiation, via the down-regulation of non-neuronal transcripts, thereby helping to establish neuronal cell identity [11], [12]. Involvement of miRNAs in the maturation of post-mitotic neurons has also been demonstrated [13], . Although most previous studies focused on single miRNAs, it is now thought that at least 100 miRNAs present in postmitotic neurons contribute to fine-tuning of dendritic protein levels in response to different patterns of synaptic activation [16]. One final, but very important feature of miRNAs is their ability to be transferred between cells via specific transporters, thus potentially effectuating the regulation of gene expression in neighboring cells [17].

Here, we present experimental genome-wide analyses of gene expression, and predictive bioinformatics analyses, which suggest that miRNAs may contribute to the co-regulation of gene expression in different cell types (i.e., in neurons, astrocytes and oligodendrocytes) at different postnatal ages in distinct regions of the developing monkey hippocampus. Interestingly, sixty-five percent of these predicted miRNAs are conserved across species, from rodents to humans; whereas thirty-five percent are specific to primates, including humans. In addition, we found that some genes exhibiting greater down-regulation of their expression were the predicted targets of a greater number of miRNAs.

## Materials and Methods

### Experimental Subjects

Sixteen male rhesus monkeys (*Macaca mulatta*; four 1-day-olds, four 6-month-olds, four 1-year-olds and four 6–12-year-olds) were used for this study. These animals were used in a previously published study [7]. Monkeys were injected with an overdose of sodium pentobarbital (50 mg/kg i.v., Fatal-Plus, Vortech Pharmaceuticals, Dearborn, MI) and the brain rapidly extracted. Five-millimeter thick slices of the brain were cut and stored overnight in RNAlater™ (Ambion, Austin, TX) at 4°C. Brain slices were then frozen in liquid nitrogen and re-sectioned at 100 µm for microdissection. Five hippocampal regions were microdissected, including the entorhinal cortex (all layers at the mid-rostrocaudal level; intermediate division, Ei), and all layers of the dentate gyrus, CA3, CA1 and subiculum at mid-rostrocaudal level of the body of the hippocampus (at the level of the lateral geniculate nucleus). Only the mid-transverse portion of each region was dissected to ensure the specificity of the sample. RNA was isolated with Trizol® and single stranded cDNA synthesized starting with 10 µg of total RNA. The sample from each region from each monkey was run on a separate chip, thus totaling 4 (animals per age) × 4 (ages) × 5 (regions)  = 80 independent chips.

### Microarray Platform

Microarray analysis was performed using the GeneChip® Human Genome U133 Plus 2.0 and the Two-Cycle Target Labeling and Control Reagents kit (Affymetrix, Santa Clara, CA) according to the manufacturer’s protocol. Following the first round of amplification with the MEGAscript T7 kit (Ambion, Austin, TX) and synthesis of single stranded cDNA, a second round of in vitro transcription was carried out using the IVT labeling kit (Affymetrix, Santa Clara, CA). U133 Plus 2.0 arrays were scanned on a GeneChip Scanner 3000.

Analyses of gene expression levels were performed with ArrayAssist® Expression Software 4.0 (Stratagene, La Jolla, CA) and a custom-made script for repeated measures ANOVA. Probe-level analysis was performed with the Robust Multichip Averaging (RMA) algorithm. Data were log2 transformed and an analysis of variance (ANOVA) was performed in order to identify genes with differential expression in distinct hippocampal regions at different ages (statistically significant interaction between ages and regions). Adjusted p-values for multiple comparisons were calculated with the false discovery rate method of Benjamini and Hochberg [18]. Follow-up analyses comparing gene expression between two ages within individual regions were performed with two-tailed unpaired t-tests. SigmaStat 3.5 statistical software was used for these analyses (Systat Software Inc.). Significance level was set at p<0.05, unless specified otherwise.

### miRNA Identification – Target Analysis

We used seven different miRNA databases (microRNA.org, hsa-TargetScan, mml-TargetScan, miRDB, hsa-miRBase, mml-miRBase, TarBase v5) to determine the predicted targets of known miRNAs. However, the lists of miRNA target genes obtained with TarBase v5, mml-miRBase and miRDB were insufficient to allow large-scale analyses and are therefore not presented here.

#### microRNA.org (Homo sapiens)

microRNA.org is a comprehensive resource of predicted miRNA targets and expression profiles. Target predictions are based on the miRanda algorithm which incorporates current biological knowledge on target rules and an up-to-date compendium of mammalian miRNAs [19]. We used the August 2008 release of the microRNA database (http://www.microRNA.org/microrna/getDownloads.do). The miRanda algorithm recognizes the importance of seed binding but does not require perfect seed complementarity (i.e., perfect hybridization between the 3′UTR of the mRNA and the 5′ end of the miRNA); it does, however, consider 3′-miRNA compensatory target sites. miRanda requires conservation of target site position on the mRNA between human and rodent with 90% identity [19].

#### hsa-TargetScan (Homo sapiens) and mml-TargetScan (Macaca mulatta)

TargetScan is a web resource that predicts biological targets of miRNAs by searching for the presence of 8mer and 7mer sites that match the seed region of each miRNA [20]. We used version 5.1 of April 2009 for the miRNAs predicted gene target list of TargetScan (http://www.targetscan.org/cgi-bin/TargetScan/data_download.cgi?db=vert_50). Without a cut-off, TargetScan predicts a target simply by virtue of the presence of at least one 7-nucleotide seed match to the miRNA in orthologous UTRs of each of five different species but can also detect non-conserved sites; TargetScan then focuses on the perfect complementarity of 5′ target sites [20].

#### miRDB (Homo sapiens)

miRDB is an online database for predicted miRNA targets in animals [21], [22]. We used the predicted targets of miRDB Version 3.0 of April 2009.

#### hsa-miRBase (Homo sapiens) and mml-miRBase (Macaca mulatta)

miRBase is a web resource containing computationally predicted targets for miRNAs across many species [23]. We used the miRBase Targets Release Version v5 (http://www.ebi.ac.uk/enright-srv/microcosm/cgi-bin/targets/v5/download.pl). The core predictions of miRBase are generated “in-house” using the miRanda algorithm [23], [24], [25]. In the hsa-miRBase, the most highly significant probability values tend to represent miRNA–target interactions that are conserved across multiple species. Strict complementarity at the seed region is required and misalignment of more than one base leads to the rejection of the potential target [23], [24], [25].

#### TarBase v5.0

Tarbase is a database with more than 1,300 experimentally supported miRNA target interactions [26]. We used the June 2008 release.

Interestingly, the manner by which these algorithms identify miRNA targets, i.e., through target conservation between species, does not prevent the identification of potential regulators of the 1000 down group that are species-specific. Indeed, the fact that a target is present in many species does not imply that the corresponding miRNA is expressed in all of these species.

### Functional Analyses

We used the DAVID (Database for Annotation, Visualization and Integrated Discovery) functional annotation tool [27], [28] to perform functional analyses of particular gene groups. DAVID provides the number of genes for each enriched functional term in a given list of genes, the percentage of total genes involved, and calculates a modified Fisher Exact p-value comparing the number of genes included in a functional category and the number of genes present in a control list (genes randomly chosen in the Affymetrix GeneChip® Human Genome U133 Plus 2.0 by DAVID). When members of two independent groups can fall into one of two mutually exclusive categories, the Fisher Exact test is used to determine whether the proportion of those falling into each category differs by group. In the DAVID annotation system, the Fisher Exact test is adopted to measure the gene-enrichment in annotation terms.

## Results

### Microarray Analysis of Gene Expression

**Figure 1:**
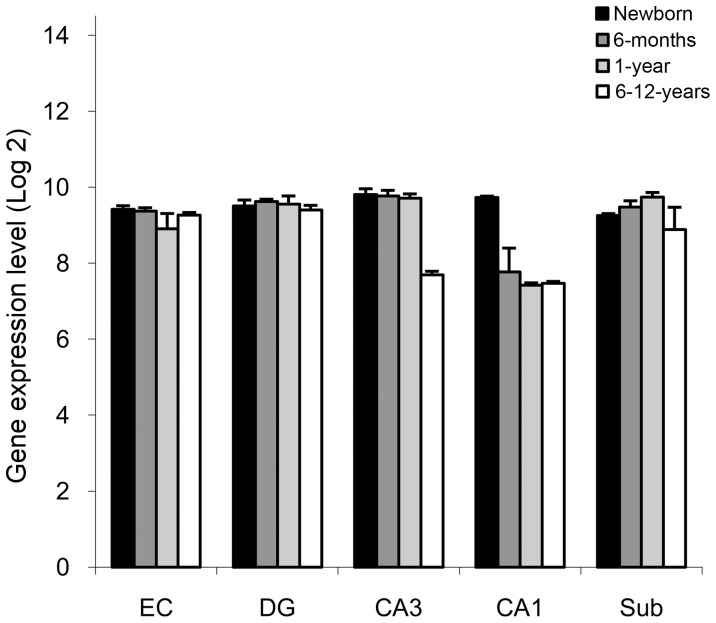
Gene expression pattern of down-regulated genes. Probeset *CALM3-200622_x_at* specific for the gene CALML2, indicated a down-regulation of the target RNA. This probeset is representative of the pattern observed in the 1000 down and down-noP gene groups (see main text for details). Differences in gene expression observed between one year of age and adulthood in CA3, and between birth and six months of age in CA1 were statistically significant (ANOVA: interaction ages x regions, Benjamini-Hochberg correction p<0.05; unpaired t-test (ages within each region), CA3: newborn, 6-month, 1-year >6–12-year; CA1: newborn >6-month, 1-year, 6–12-year, all p<0.05). Each bar represents the mean (+/− SEM) of the gene expression of the four animals of the same age. See also **Table S1** and **Table S2**.

We first identified genes exhibiting specific changes in expression at different ages in distinct regions of the monkey hippocampal formation. In addition to the previously identified group of genes expressed in astrocytes [7], we found a number of genes expressed in neurons and oligodendrocytes that exhibited the same patterns of expression. Specifically, we identified 1755 genes exhibiting a decrease in expression from birth to six months of age in CA1, and after one year of age in CA3, to reach the expression levels observed at 6–12 years of age (**Figure 1**; ANOVA interaction ages X regions: FDR corrected p<0.05; CA1: newborn >6-month, 1-year, 6–12-years, unpaired t-tests, all p<0.05; CA3: newborn, 6-month, 1-year >6–12-years, unpaired t-tests, all p<0.05; Note that each of the 1755 genes exhibited both an early decrease of expression in CA1 and a later decrease in expression in CA3). Note also that the expression patterns for a subset of these genes and associated cellular processes were confirmed with RT-PCR, immunohistochemistry and electron microscopy methods [7]. From this list of 1755 genes, we excluded all genes whose function was not clearly described and were therefore often not considered adequately in miRNA-related databases. Specifically, all gene symbols containing FLJ, LOC, MGC, KIAA, DKFZP and corf were excluded, which resulted in a list of 1429 genes. After eliminating an additional 218 genes that did not have clear annotations in miRNA-related databases, we identified the 1000 genes that exhibited the highest probability of differential expression across ages in CA3 and CA1 (ANOVA interaction ages X regions: FDR corrected P<0.008665); we denoted these 1000 genes as the “1000 down” group **(Table S1)**.

We also considered a larger group of down-regulated genes, which included every gene with at least one probeset (some genes are represented by several probesets on the Affymetrix U133 Plus 2.0 microarray) exhibiting decreased expression from birth to six months of age in CA1, and from one year to 6–12 years of age in CA3, irrespective of the level of statistical significance. As before, we excluded all probesets targeting genes whose function was not clearly described. We denoted these genes as the “down-noP” group (**Table S1**), which was used for some of the gene function analyses (see below).

We also considered genes that exhibited other changes in expression in order to create comparison groups for the bioinformatics analyses. We created a control group that excluded all probesets exhibiting decreased expression from birth to six months of age in CA1, and from one year to 6–12 years of age in CA3, irrespective of statistical significance. We further excluded, from this group, all probesets exhibiting the reverse pattern, as these probesets might actually hybridize precursors of miRNAs targeting the down-regulated genes (not shown), rather than genes exhibiting actual increases in expression. As was the case for the group of down-regulated genes, we also excluded all probesets corresponding to the symbols FLJ, LOC, MGC, KIAA, DKFZP and corf. Finally, we randomly selected 1000 genes that were present in the miRNA-related databases; we denoted these 1000 genes as the “1000 control” group (**Table S1**).

### Bioinformatics Analysis of miRNA– Target Gene Interaction

We used several bioinformatics tools, including NCBI (National Center for Biotechnology Information), miRBase, TargetScan, microRNA.org and Affymetrix bioinformatics software, to perform predictive analyses and determine possible mechanisms of co-regulation of the expression of these genes expressed in different cell types. Our analyses specifically considered the involvement of miRNAs (small RNA molecules acting as post-transcriptional regulatory elements), which have been shown to play a major role in developmental processes [9], [10]. We analyzed each known miRNA (listed in any of the four miRNA databases; microRNA.org, hsa-miRBase, hsa-TargetScan, mml-TargetScan) and its predicted targets in order to identify all of the miRNAs that might preferentially target any of the down-regulated genes. First, we compared statistically the number of genes included in the group of 1000 down-regulated genes versus the 1000 control group, which were predicted targets of all miRNAs listed in the four databases. We repeated the miRNA target analysis with two other groups of down-regulated genes and three other groups of control genes (data not shown), which confirmed the results presented below. Second, we integrated and compared the results of the analyses derived from the four independent databases in order to most thoroughly and reliably identify miRNA candidates [25] that might be responsible for regulating the 1000 down genes.

To perform this analysis, we produced lists of all miRNAs targeting genes of the 1000 down group, or genes of the 1000 control group based on the four databases (microRNA.org, hsa-miRBase, hsa-TargetScan, mml-TargetScan). We determined how many genes are targeted by every single miRNA in each group, and identified which miRNAs preferentially target the down-regulated genes (1000 down group), as compared to the control genes (1000 control group; Chi-square goodness of fit analysis, p<0.05). The microRNA.org database revealed 260 miRNAs targeting more genes in the 1000 down group than in the 1000 control group; the hsa-miRBase database revealed 115 miRNAs; the hsa-TargetScan database revealed 27 miRNAs, and the mml-TargetScan database revealed 16 miRNAs (**Table S2**; Note that we found no differences in UTR length between the 1000 down and 1000 control groups). Differences in the numbers of miRNAs identified in the different databases were due to the different assumptions, algorithms or parameters used to predict the correspondence between specific miRNAs and their target genes (see Materials and Methods for details).

**Figure 2:**
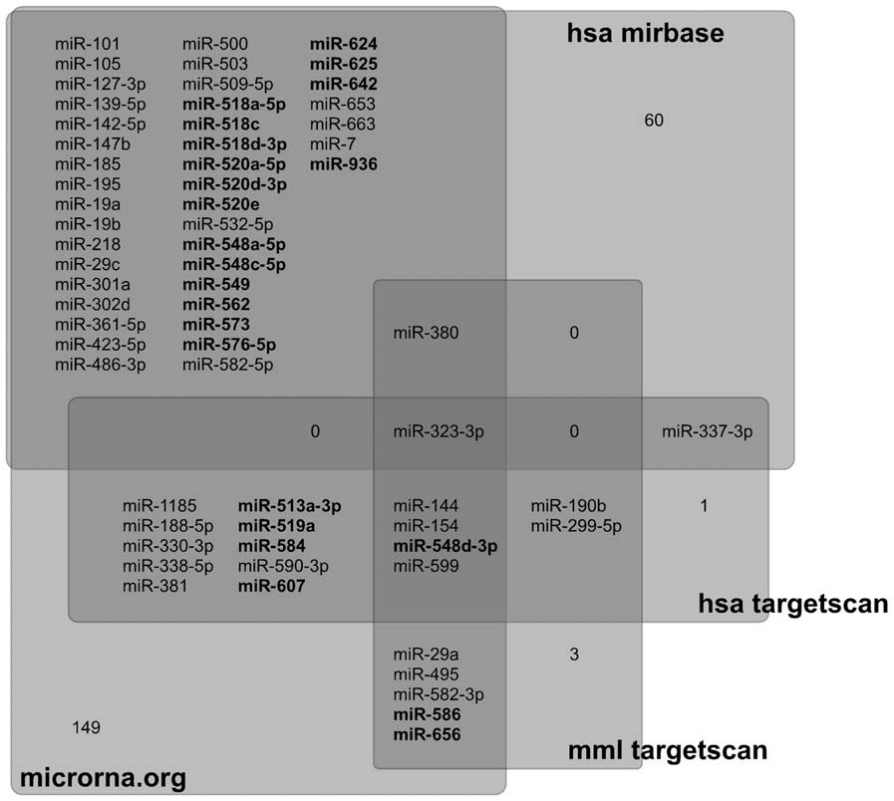
miRNAs targeting the down-regulated genes. miRNAs present in *Macaca mulatta* that preferentially target down-regulated genes, as predicted by four different databases: microRNA.org, hsa-miRBase, hsa-TargetScan and mml-TargetScan (see main text for details). 65 miRNAs were predicted by at least 2 databases. Primate-specific miRNAs are identified in bold. See also **Table S2**. The numbers (1, 3, 60, 149) represents the numbers of miRNAs identified as potential regulators of the down-regulated genes in only one database, which were not considered in subsequent analyses.

We found one miRNA, miR-323-3p, which preferentially targets the down-regulated genes and was identified in all four databases (**Figure 2** **; Table S2**). miR-323-3p is conserved across species, from rodents to humans. There were five other miRNAs identified in at least three databases: miR-144, miR-154, miR-380, miR-548d-3p, and miR-599. Interestingly, miR-548d-3p is a member of a miRNA family present only in primates. Moreover, although the miR-548 miRNA family has been identified in *Homo sapiens*, *Pan troglodytes*, *Pongo pygmaeus* and *Macaca mulatta*, the miR-548d members present the particularity of being present only in *Homo sapiens* and *Macaca mulatta*. Although we can be very confident about the six miRNAs predicted by at least three databases, this analysis might be too restrictive for a predictive analysis. We therefore considered the predictions common to at least two databases in order to increase our ability to identify the miRNAs that might regulate the expression of the 1000 down group of genes; we found 78 miRNAs predicted by at least two databases (**Table S2**).

According to the miRBase [23] and miRviewer [29] databases, 65 of these 78 miRNAs are expressed in *Macaca mulatta* (**Figure 2** **; Table S2**). According to miRBase, amongst these 65 miRNAs, 33 miRNAs (51%) are included in miRNA clusters, i.e., grouped in genomic locations where they are susceptible to be co-regulated; 23 miRNAs (35%) are primate-specific; and 18 miRNAs (28%) are not included in clusters and are not primate specific. We used miRBase and UCSC-Blat to determine the genomic location of every miRNA listed for *Macaca mulatta*. We found that seven miRNAs are included in the miR379-410 cluster, one of them being primate specific (miR-656). Seven miRNAs are included in the C19MC cluster, which is a primate-specific cluster on chromosome 19 (miR-518a-5p, miR-518c, miR-518d-3p, miR-519a, miR-520a-5p, miR-520d-3p, miR-520e). miR-513a-3p and miR-509-5p are included in another primate-specific cluster on chromosome X.

### miRNA Additive Effect

**Figure 3:**
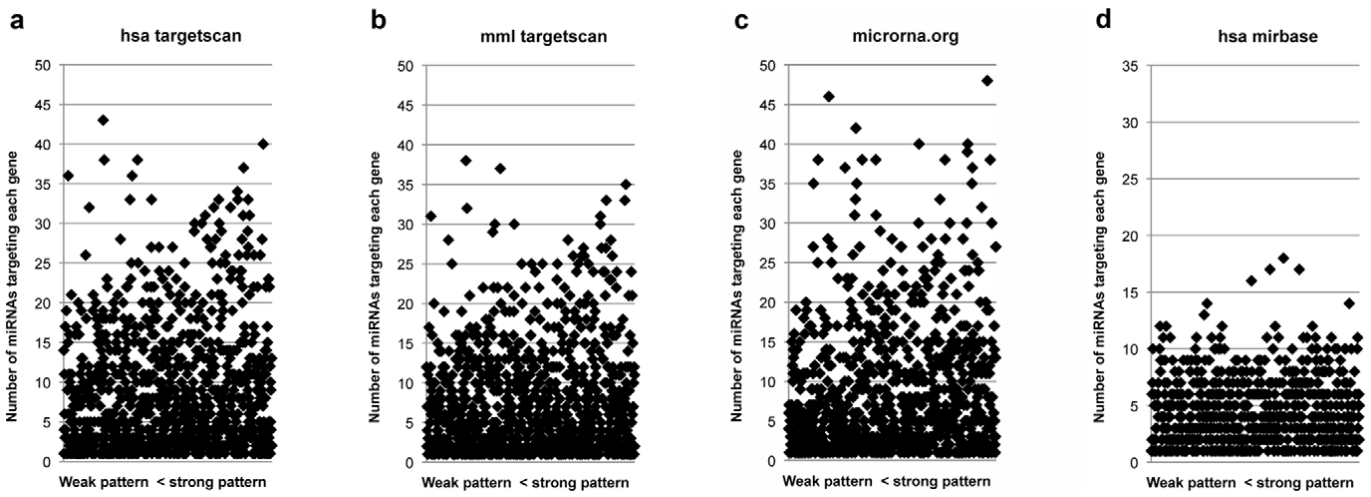
Number of miRNAs and amplitude of gene regulation. Graphical representation of the relationship between the amplitude of the change in gene expression observed in CA1 (x axis) and the number of miRNAs targeting each gene (y axis). Each point represents a gene of the 1000 down group. **a.** hsa-TargetScan. **b.** mml-TargetScan. **c.** microRNA.org. **d.** hsa-miRBase. See also **Table S3**.

In order to determine whether genes targeted by multiple miRNAs were more common in the 1000 down group than the 1000 control group, we also identified the genes included in the 1000 down and 1000 control groups that were targeted by one or more of the 65 miRNAs present in *Macaca mulatta* previously identified. We then determined how many miRNAs were targeting the same gene. First, we found differences between the number of genes targeted by multiple miRNAs between the 1000 down and 1000; control group (**Table 1**). Second, we also found that the degree to which some genes are down-regulated is positively correlated with the number of miRNAs that target those genes (**Figure 3**, **Table S3**). Specifically, we calculated the difference in the mean expression level of a given gene in CA1 between newborn and adult monkeys. The greatest differences were characterized as a strong change in expression, and the smallest differences as a weak change in expression. We then compared the number of miRNAs targeting either the 200 or the 500 genes exhibiting the strongest changes in expression (the group of 200 exhibited differences between 1.51 and 3.91, the group of 500 exhibited differences between 1.0517 and 3.91) with the number of miRNAs targeting either the 200 or the 500 genes exhibiting the weakest changes in expression (the group of 200 exhibited differences between 0.2 and 0.7, the group of 500 exhibited differences between 0.2 and 1.0515). We used the microRNA.org, hsa-miRBase, hsa-TargetScan and mml-TargetScan databases to determine statistically significant differences (Chi-square goodness of fit, p<0.05).

**Table 1:** Numbers and proportions of target genes of the 65 miRNAs identified by at least two databases and present in *Macaca mulatta*, which are comprised in the 1000 down group versus the 1000 control group, as a function of the number of miRNAs targeting each target gene.

Number of miRNAs targeting the same gene	1	2	5	10	15	20	25	30	35
Number of genes targeted in 1000 down (d)	853	758	534	306	175	92	48	25	16
Number of genes targeted in 1000 control (c)	778	629	384	191	86	47	21	8	3
Ratio d/c	1.1	1.21	1.39	1.6	2.03	1.96	2.29	3.13	5.33
Chi-square significance, p<0.05	No	Yes	Yes	Yes	Yes	Yes	Yes	Yes	Yes

Analysis performed with the microrna.org database.

Altogether, these results suggest that the regulation of the expression of genes included in the 1000 down group involves the combined influence of several miRNAs, rather than the impact of individual miRNAs on individual genes, and support the findings of others suggesting that, *in vivo,* developmental biological processes typically involve a combination of different miRNAs rather than single miRNAs [30].

### Global Functional Analysis

We used the DAVID functional annotation tool [27], [28] to perform a functional analysis of the genes exhibiting a down-regulation of their expression from birth to six months of age in CA1, and after one year of age in CA3, irrespective of statistical significance (6894 genes; **Table S1)**. We found three main categories highlighted by the KEGG pathway analysis (**Table S4A**): (1) the mitogen-activated protein kinase (*MAPK*) signaling pathway, (2) the production and degradation of proteins, and (3) the RNA processing pathway. As the *MAPK* signaling pathway is highly studied and thus linked to many different functional pathways that might not be directly relevant to hippocampal development, we evaluated the more specific, individual pathways listed in KEGG: we identified the long-term potentiation (LTP) and long-term depression (LTD) pathways as functional groups containing *MAPK* pathway-involved molecules, whose relation with hippocampal processes such as synaptic plasticity is therefore obvious [31]. The neurotrophin signaling pathway, adherens junction and axon guidance functional terms all implicate *MAPK* pathway molecules in relation to the actin cytoskeleton, suggesting that morphological plasticity is also concerned. The insulin-signaling pathway was likely identified because of its influence on the *MAPK* cascade. The fact that endometrial cancer, renal cell carcinoma, pancreatic cancer, colorectal cancer and non-small cell lung cancer were identified as significant functional terms was also likely due to the fact that *MAPK* pathway molecules are present in all of these cancer pathways. The functional terms ubiquitin mediated proteolysis, proteasome, lysosome, lysine degradation, valine, leucine and isoleucine degradation are all linked to protein degradation; whereas the aminoacyl-tRNA biosynthesis, basal transcription factors and pyruvate metabolism terms are linked to protein or amino-acid production. Amyotrophic lateral sclerosis and Huntington’s disease were identified because their pathogenic mechanisms involve protein degradation. The spliceosome and RNA degradation functional terms clearly implied a change in RNA processing.

Second, we analyzed the 1000 down group of genes with the DAVID functional annotation tool and highlighted 78 enriched Gene Ontology (GO) terms related to biological process with a P-value <0.005 (**Table S4B,C**). In accordance with the results of the KEGG pathway analysis, we identified three main functional groups of genes among the GO terms. Twelve of the GO terms were related to the *MAPK* pathway, 29 were related to production and degradation of protein and 11 to RNA processing and transcription. The other enriched GO terms were related to development, differentiation, or the regulation of axons and cytoskeleton. The more restrictive analysis of the 1000 down gene list thus confirmed the most important functional groups found in the analysis of all down-regulated genes.

### Cell-specific Functional Analysis

In a previous study [7], we identified a number of genes that were related to astrocyte structure and function which exhibited, in CA1, a down-regulation of their expression from birth to adulthood. To further evaluate the expression of genes specific to different cell types, we subdivided the 1000 down gene group based on a list of cell-specific genes derived from a mouse transcriptome study [32]. That study identified genes significantly enriched by at least 1.5-fold in astrocytes (2618 genes), neurons (2036 genes) and oligodendrocytes (2228 genes). Based on this list, we found 157 genes enriched in astrocytes, 132 genes enriched in neurons and 132 genes enriched in oligodendrocytes in the 1000 down group of genes (**Table S1**). KEGG and GO analyses performed on the three lists of genes enriched in different cell populations revealed common, as well as distinct functional pathways.

#### Neurons

Five biological process-related GO terms were identified with high statistical value (P<0.005) among the 132 genes enriched in neurons contained in the 1000 down gene list: synaptic transmission, transmission of nerve impulse, regulation of neuronal synaptic plasticity, ion transport, and generation of precursor metabolites (**Table S4D**). Not surprisingly, the genes in the GO terms groups were related to glutamate and GABA transmission, to the *MAPK* pathway and to Ca^2+^ signaling pathways. The genes related to ion transport probably mediate a change in neuron excitability. Some genes were linked to dendrite development and vesicle-mediated transport.

#### Oligodendrocytes

The most significant GO terms (P<0.005) among the 132 oligodendrocytes-enriched genes were related to myelin and lipid metabolism, RNA processing and intracellular transport (**Table S4E**).

#### Astrocytes

Many biological process-related GO terms were identified in the 157 genes enriched in astrocytes with high significance value (P<0.005). These GO terms were mainly related to cell cycle, regulation of proliferation, differentiation and development. Interestingly, GO terms related to morphogenesis, neurogenesis and axogenesis were also shown to be significant (**Table S4F**).

#### No specificity

554 genes of the 1000 down group that were not preferentially enriched in any particular cell type were identified as being involved in two of the same processes highlighted in the whole 1000 down group analysis, i.e., RNA processing and transcription, and protein production and catabolism. The *MAPK* signaling pathway and its LTP- and LTD-related genes were preferentially down-regulated only in neurons. But the RNA processing, and the protein production and catabolism functional groups were equally regulated in the three different cell types (**Table S4G**).

As described above, the co-regulation of such a large number of genes in different cell types is likely based on a regulatory mechanism including miRNAs. Interestingly, the gene *SIDT2*, which codes for a membrane channel that allows the transmission of sequence-specific RNA interference molecules between cells [33], exhibited an up-regulated pattern of expression that mirrored that of the down-regulated genes (not shown). Importantly, the probeset *SIDT2*-56256_at, indicating an up-regulation of the target RNA, hybridized with an untranslated region of the *SIDT2* gene in the sense direction. It is therefore possible that its increased expression at different postnatal ages allows the facilitated transmission of miRNAs through the membrane of different cell types, possibly explaining how miRNAs can contribute to the co-regulation of genes enriched in different cell types during development.

### Functional Analysis of Preferential Targets

In order to determine which genes might be particularly important for the development of the primate hippocampus, we performed a functional analysis of genes that were identified as preferential targets of miRNAs. First, we identified the genes that were preferentially targeted by the 65 miRNAs predicted by at least two databases and present in *Macaca mulatta*. We identified 195 genes from the 1000 down group that were predicted targets of at least ten miRNAs in at least three databases (**Table S1**). Analysis of these genes revealed that the overrepresented GO terms were related to RNA processing, transcription regulation, and protein production and degradation (**Table S4H**). *MAPK* signaling pathway did not appear to be preferentially targeted. Interestingly, genes specific for different cell types were preferential targets of these 65 miRNAs. Indeed, among the 195 genes targeted by at least ten miRNAs, 35 genes were enriched in astrocytes (including *GFAP*, glial fibrillary acidic protein), 35 were enriched in oligodendrocytes (including *MOBP*, myelin associated oligodendrocyte basic protein) and 36 were enriched in neurons (including *NRXN3*, cell surface protein neurexin 3).

Second, we evaluated whether certain genes were preferentially targeted by different groups of miRNAs. We determined how many primate-specific miRNAs among the 65 miRNAs targeted each gene of the 1000 down list, using four different databases (microRNA.org, hsa-TargetScan, mml-TargetScan and hsa-miRBase). We identified 23 genes as preferential targets of primate-specific miRNAs in at least 3 databases (**Table S1**). Analysis of these 23 genes revealed that some of them were involved in translation regulation, i.e., the same functional role as the whole group of down-regulated genes (**Table S4I**). Among the 23 primate-specific target genes, the seven genes enriched in astrocytes (*BMPR1A*, *CHST11*, *CLIC1*, *FARS2*, *HMGN1*, *PTS*, *SOX2*) were involved in proliferation and differentiation, the two genes enriched in neurons (*GNB5*, *CTNNA2*) were involved in dendritic morphology and neuron excitability and the gene enriched in oligodendrocytes (*LASS2*) was involved in myelination. All these genes might therefore be differentially regulated by primate-specific miRNAs and thus underlie some subtle differences in the structural and functional properties of individual cell types and hippocampal circuits observed between species.

## Discussion

Our predictive bioinformatics analyses suggest that miRNAs may contribute to the regulation of gene expression during early postnatal development of the monkey hippocampus. We identified 65 miRNAs present in humans and monkeys as the primary regulators of the expression of a group of more than 1000 genes exhibiting a down-regulation of their expression, from birth to six months of age in CA1 and after one year of age in CA3, to reach levels observed at 6–12 years of age. Importantly, these 65 miRNAs may contribute to the down-regulation of the expression of different groups of genes enriched in astrocytes, neurons and oligodendrocytes, thus providing a fundamental means for the co-regulation of gene expression in different cell types. Note that the expression of ten of the 65 miRNAs we identified in the current study (miR-101, miR-127-3p, miR-139-5p, miR-142-5p, miR-185, miR-195, miR-218, miR-29a, miR-29c, miR-381) has been detected in samples of adult human hippocampus containing different subregions [34]. Our current results suggest that a larger number of miRNAs are expressed in distinct hippocampal regions at different times during postnatal development.

Most of these 65 miRNAs are present in clusters or are primate-specific. In addition, the observed down-regulation of gene expression from birth to adulthood may result from the combined effect of a number of different miRNAs, as some genes exhibiting stronger down-regulation of their expression are the predicted targets of a greater number of miRNAs. We first consider different aspects of the regulation of gene expression by miRNAs: the importance of primate-specific miRNAs, the regulation of miRNA expression, the additive effect of multiple miRNAs, and the transmission of miRNAs between different cell types. We then discuss the possible functional implications of our findings for the maturation of specific cellular processes in the normally developing hippocampus.

### Primate-specific miRNAs

Thirty-five percent of the miRNAs identified as regulators of the 1000 down group of genes are primate-specific [29]. These miRNAs could contribute to some of the structural and functional differences observed between primate and non-primate mammals. For example, the differences in dendritic morphology of CA1 neurons observed between rats and monkeys [35] could be related to the regulation of genes like *CTNNA2* (coding for the protein alpha-N-catenin), whereas *GNB5* (coding for the guanine nucleotide-binding protein, beta-5) regulation may produce differences in the electrophysiological characteristics of hippocampal neurons between these two species. Indeed, these genes were predicted to be preferentially targeted by primate-specific miRNAs and are involved, respectively, in dendritic morphology and the regulation of the stability of synaptic contacts [36], and in the electrophysiological properties of neurons [37].

Other differences found among primate species might be related to the further evolution of these primate-specific miRNAs. Indeed, the numbers of miRNAs of the miR-548 and C19MC families increase from *Macaca mulatta* and *Pongo pygmaeus*, to *Pan troglodytes* and *Homo sapiens*, via homolog or partially homolog replication (transposable elements) [29], [38]. Such species differences in miRNAs might lead to subtle differences in the regulation of gene expression that might underlie species differences in hippocampal structure and function that have emerged over the course of evolution. These differences might be particularly important to consider when extrapolating from experimental results in model animals, such as rodents and monkeys, to clinical applications in humans.

Primate-specific miRNAs appear necessary to achieve the precise regulation of gene expression that contributes to the normal development of the monkey hippocampus. To date, however, the 23 primate-specific miRNAs that we identified as regulators of the 1000 down group of genes have not yet been shown to be present in the adult primate hippocampus. Indeed, only one study evaluated the expression of miRNAs in the adult human hippocampus, and used samples that included portions of several fields [34]. As our own analyses have shown, the regulation of gene expression is both region- and time-specific. A global analysis mixing the different regions from adult subjects is thus unlikely to detect specific miRNAs contributing to the regulation of gene expression in distinct hippocampal regions during development. Moreover, the statistical analyses used in [34] were biased towards the detection of highly expressed miRNAs, and their methods exhibited a high degree of variability in the measurements of the expression of low-abundance miRNAs. Now that we have predicted that a number of miRNAs might act together to regulate gene expression at different ages in the primate CA1 and CA3, it will be possible to target these miRNAs more specifically in future experimental studies.

### Regulation of miRNA Expression

#### Clusters

Among the 65 miRNAs identified as potential regulators of the 1000 down gene group, seven belong to the miR-379-410 cluster (also named C14MC, based on its location on *Homo sapiens* chromosome 14). The miR-379-410 cluster is located on chromosome 7 in *Macaca mulatta.* It comprises more than 50 miRNAs and its expression is induced by neuronal activity [30]. The miR-379-410 cluster is expressed in rodents and primates, and might subserve the same function in different species. Among the seven miRNAs belonging to this cluster that we identified in this study, miR-323-3p has been shown to be expressed in the mouse hippocampus and to play a role in learning, memory, anxiety and exploratory behaviors [39]. miR-381, another one of the seven miRNAs belonging to the miR-379-410 cluster identified in this study, has been shown to be involved in the regulation of dendritic outgrowth of hippocampal neurons controlled by neuronal activity [30]. Interestingly, miR-381 is also expressed in the adult human hippocampus [34]. It thus stands to reason that the growth of dendrites might be regulated by the miR-379-410 cluster of miRNAs, which are dependent on neuronal activity for the regulation of their expression. Indeed, we previously reported developmental patterns of volumetric changes [6] and protein expression [7] that are concomitant with the developmental patterns of gene expression observed in CA1 and CA3 (current study; [7]). The role of the miR-379-410 cluster in hippocampal development should be the subject of future experimental studies.

#### Neuronal activity

miR-379-410 expression is also induced by neuronal activity via the *MEF2* transcription factor [30], whose activity is induced by calcium influx through NMDARs and L-type VGCCs in hippocampal neurons [40]. Most interestingly, Altier and colleagues [41] have recently reported direct ubiquitination-mediated degradation of L-type voltage-gated calcium channel subunit Cav1.2. In our study, we identified genes involved in ubiquitin-mediated proteolysis to be particularly numerous in the down-regulated group of genes. This suggests new avenues for future investigations of the upstream mechanisms that might regulate neuronal excitability and synaptic transmission in distinct CA3 and CA1 circuits at different postnatal ages.

#### Imprinting

Other regulatory mechanisms, like imprinting, might also contribute to the regulation of expression of the miR-379-410 and C19MC clusters. Indeed, although these two clusters are evolutionary distinct (the miR379-410 cluster is conserved among eutherian species (placental mammals) and the C19MC cluster is primate-specific), their expressions are both susceptible to regulation via imprinting mechanisms [38], [42]. miR379-410 derives from the maternally inherited chromosome and C19MC from the paternally inherited chromosome. The regulation of expression of these two imprinted clusters, although not fully understood, likely involves DNA methylation and long non-protein-coding RNAs (ncRNAs) also known as imprinted macroRNAs [38], [42]. If this hypothesis is verified experimentally, it would provide a new perspective to consider when searching for fundamental mechanisms underlying the subtle regulation of gene expression in the developing hippocampus. It might also explain how some epigenetic factors could contribute to the etiology of neurodevelopmental disorders affecting this brain region in humans.

### Additive Effect of Multiple miRNAs

Our study is also the first to reveal a significant, positive correlation between the number of miRNAs targeting a group of genes and the degree of down-regulation of these genes. Our experimental data therefore support the conclusions of earlier studies suggesting the importance of multiple miRNAs targeting the same gene [43]. Importantly, we evaluated this fundamental mechanism of regulation of gene expression in a normally developing system. Thus, many miRNAs might act in concert to regulate the expression of a large ensemble of genes involved in the postnatal, structural and functional maturation of the primate hippocampal formation. Interestingly, although the additive effect of multiple miRNAs was statistically significant in three different databases, the effect was stronger and more easily detectable with the hsa-TargetScan and mml-TargetScan databases (**Figure 3** **; Table S3)** than with the microRNA.org database (the effect was not significant with hsa–miRBase). The assumptions and the algorithms used in the different databases might explain these differences and support additional hypotheses regarding the biological mechanisms of RNA interference. Indeed, a high or perfect homology between miRNA and its corresponding mRNA targets often leads to the cleavage of the mRNA molecule, whereas a weaker homology between a miRNA and its target mRNA is more likely to lead to the inhibition of the translation of the targeted mRNA without cleavage of the molecule [44]. In our microarray data, cleavage should be detected as a decrease in mRNA level, whereas an inhibition of the translation would remain undetected because mRNA level would not change. In accordance, as TargetScan target prediction insists more on a high homology between the 5′ seed region of the miRNA and its mRNA target, it might be more sensitive to detect mRNA targets exhibiting decreased expression; in contrast, microRNA.org and hsa–miRBase, which are more permissive for 5′ seed partial match and compensate with 3′ complementarity, are more likely to identify mRNA targets that have not undergone cleavage and are therefore not detected as exhibiting a decreased mRNA expression level with the microarray platform. Nevertheless, microRNA.org also revealed the miRNA additive effect.

It is interesting to note that sixteen genes, *AKAP13*, *CAMTA1*, *CNOT6L*, *FBXW2*, *HNRPA3*, *HS2ST1*, *IMPAD1*, *MAPK1*, *NAV1*, *NRXN1*, *NSL1*, *PRDM2*, *PTEN*, *TBL1XR1*, *WDFY3*, *ZFP91* which represent 1.6% of the 1000 down group of genes, did not follow the trend revealed in the TargetScan and microRNA.org databases (**Table S3**). They were highly targeted by miRNAs but exhibited only weak, although significant, patterns of down-regulation. These mRNAs might undergo posttranscriptional processing in their UTR 3′ end, which could partially prevent the miRNAs from hybridizing them and thus result in a down-regulation of expression that is weaker than expected based on the high number of miRNAs targeting them [45].

### miRNA Transmission between Different Cell Types

Among the 1000 down gene group, we identified 157 genes enriched in astrocytes, 132 in neurons, and 132 in oligodendrocytes. The transport of RNA interference molecules between cells via dedicated RNA transporters [33] might contribute to the co-regulation of expression of hundreds of genes in different cell types. Indeed, cultured mouse and human cells are able to import siRNA (exogenous, small interfering RNA molecules structurally similar to mature endogenous miRNAs) via specialized channels. More specifically, the over-expression of the double-stranded RNA (dsRNA) transporter channel, SID-1, improves the ability of cultured human cells to import siRNA, resulting in an increase in siRNA-mediated gene silencing efficacy [46]. *SIDT1* and *SIDT2* are paralogs of SID-1, and both participate in the transport of RNA interference signals into cells [33]. Our microarray data revealed that *SIDT2* exhibited an increased level of expression that mirrored the typical pattern of expression of the down-regulated genes. *SIDT2* might therefore facilitate the transport of miRNA molecules between different cell types [17] at specific ages during postnatal development.

### Functional Implications

Our study revealed that a large number of genes were down-regulated in the hippocampus (i.e., CA3 and CA1) of older mature monkeys, as compared to young developing monkeys. In this section, we consider how the specific decrease in the expression of certain functional groups might regulate synaptic plasticity and thus contribute to the emergence of more selective and efficient memory processes.

#### Protein lifespan

We have identified an important functional group involved in protein metabolism in the genes that were down-regulated with age. This group can be subdivided into two subgroups: one involved in protein synthesis and the other in protein degradation. The importance of protein synthesis in synaptic plasticity is well established. Indeed, as early as the 1960’s, researchers showed that protein synthesis inhibitors impair memory performance, as well as LTP and LTD induction in CA1 [47], [48]. Demonstration of the role of protein degradation pathways in synaptic plasticity is more recent. For example, the proteasome inhibitor lactacystin, which prevents protein degradation, also leads to impaired memory performance and decreased LTP in CA1 [49]. From these two series of experiments, it has become clear that the regulation of protein synthesis and degradation impacts synaptic plasticity as well as learning and memory processes. The down-regulation of both protein synthesis and protein degradation genes likely impacts the plasticity mechanisms that regulate synaptic transmission [50]. For example, the concomitant down-regulation of genes involved in protein synthesis and those involved in protein degradation that we have observed might reflect an increase in protein lifespan. This might, in turn, lead to more stable and longer lasting changes in the efficacy of synaptic transmission, which could be beneficial for learning and the maintenance of long-term memories.

#### Astrocytic processes

We have previously discussed how the regulation of gene expression and astrocytic processes might contribute to the emergence of selective memory function, via the regulation of glutamate concentration in the synapse by astrocytes [7]. In addition, the seven primate-specific astrocytic target genes (*BMPR1A*, *CHST11*, *CLIC1*, *FARS2*, *HMGN1*, *PTS*, *SOX2*) identified in this study were found in GO terms involved in stem cell maintenance, cell differentiation and development. Stem cell maintenance is unlikely to be involved in the regulation of gene expression for the purpose of neurogenesis in postnatal CA3 and CA1, as postnatal neurogenesis is clearly limited to the dentate gyrus [51]. However, *SOX2* has also been shown to be expressed in proliferating astrocytes after they have acquired a glial fate and until they become quiescent, and in astrocytes after injuries in order to promote gliogenesis [52]. *BMPR1A* and *FABP7* (the latter of which is not included in the 1000 down group, but is significantly down-regulated and identified as a preferential target of primate-specific miRNAs) are also involved in gliogenesis and their expression is increased following injury or ischemia [53], [54]. This suggests that the down-regulated, astrocytic genes targeted by primate-specific miRNAs might be associated with decreased gliogenesis that may be associated with a change in the cellular phenotype of astrocytes, from a proliferating state to a quiescent state. These changes could correlate with the decrease in *GFAP* gene expression and astrocytic coverage of excitatory synapses observed in CA1 between birth and adulthood [7]. As mentioned above, a number of the down-regulated, astrocytic-enriched genes targeted by the 65 miRNAs are involved in astrocytic differentiation and morphogenesis regulation. Primate-specific miRNAs included in this group could further modulate this mechanism of regulation and contribute to species differences in astrocytic morphology [55].

### Synaptic Plasticity

The functional groups of down-regulated genes preferentially expressed in neurons included genes involved in the *MAPK* signalling pathway, LTP, LTD, glutamate and GABA neurotransmitter pathways, regulation of dendritic processes and ion channels. We will consider a few of these functional groups in turn. The *MAPK* signaling pathway has been shown to be necessary for processes like LTP and is therefore essential for the mechanisms of synaptic plasticity [31]. The overexpression of plasticity-related proteins has been suggested as one possible molecular mechanism underlying some forms of autistic disorders [56]. *MAPK* pathway over-activation in transgenic mice leads to deficits in hippocampal plasticity and hippocampal-dependent learning, which correlate with an increase in GABA release in the hippocampus [31], [57], [58]. The relative over-expression of genes involved in the *MAPK* signaling pathway in young individuals, as compared to adults, might be associated with lower learning and memory abilities in young individuals. The down regulation of the expression of genes involved in the *MAPK* signaling pathway as the individual ages would thus lead to improved hippocampal function, leading to an increase in learning and memory performance normally observed during normal postnatal development [8], [59].

#### Dendritic morphology and schizophrenia

The primate-specific miRNAs targeting the neuron-enriched gene *CTNNA2*, coding for alphaN-catenin, might also contribute to the modulation of synaptic plasticity and the regulation of dendritic morphology. Extrapolating from a study in transgenic mice [36], the higher expression of *CTNNA2* in the developing hippocampus could be associated with a higher density of dendritic spines. The developmental decrease in *CTNNA2* expression could in turn contribute to the elimination of synapsin-positive spines. This hypothesis is in agreement with the concept of generalized synapse elimination during the development of the central nervous system [60]. A decrease in *CTNNA2* expression and synapse number during development could lead to an increased signal-to-noise ratio and a more efficient memory encoding system in the mature hippocampus. The fact that *CTNNA2* is a predicted preferential target of primate-specific miRNAs raises questions about species differences in synaptic plasticity, learning and hippocampus-dependent memory abilities [61]. As mentioned previously, *CTNNA2* is also a candidate gene to explain some of the morphological differences observed in CA1 pyramidal neurons between rats and monkeys [35]. Furthermore, *CTNNA2* has also been shown to be involved in schizophrenia [62]. Its implication in a human-specific disease might be linked to its regulation by primate- and even human-specific miRNAs. Indeed, although *CTNNA2* can be regulated by primate-specific miRNAs common to both monkeys and humans (miR-513a-3p, miR-518a-5p, miR-548a-5p, miR-576-5p, miR-586, miR-607, miR-625, miR-642), a number of miRNAs present in *Homo sapiens* but not in *Macaca mulatta* (miR-1208, miR-1247, miR-1290, miR-1324, miR-1825, miR-613 and miR-634) also target *CTNNA2* [19], [29]. These miRNAs might therefore contribute to a differential regulation of *CTNNA2* in humans, which could be linked to the dysregulation of gene expression associated with the emergence of schizophrenia.

#### Neuronal excitability

Finally, *GNB5* is also a predicted preferential target of primate-specific miRNAs. This gene has been shown to be involved in the GABAB-GIRK-dependent GABA-induced hyperpolarization in the mouse hippocampus [37]. Its deletion causes slower rise time and decay of IPSC and an apparently strengthened GABA-dependent behavioral change in motor activity. Its decrease through development might thus lead to a change in GABA cellular kinetics response and in a stronger response to GABA in the adult hippocampus. The fact that *GNB5* might be differentially regulated in primates, as compared to rodents, suggests the existence of potential species differences in the electrophysiological properties of hippocampal neurons. This hypothesis will require further experimental investigations.

### Conclusions

Our predictive bioinformatics analysis suggested that miRNAs may play a fundamental role in the co-regulation of gene expression in different cell types that contributes to the normal postnatal development of the primate hippocampus. This mechanism is partially conserved across species, and may thus contribute to the similarity of basic hippocampal characteristics across mammals. This mechanism also exhibits a phylogenetic diversity that might contribute to more subtle species differences in hippocampal structure and function observed at the cellular level.

## Supporting Information

Table S1 **Genes differentially expressed in CA3 and CA1 (see also** **Figure 1** **and main text for details).**
(XLS)

Table S2 **miRNAs identified as targeting more genes in the 1000 down group than in the 1000 control group.**
(XLS)

Table S3 **Relation between the number of miRNAs targeting a gene and its pattern of regulation.**
(XLS)

Table S4 **Functional analyses of down-regulated genes.**
(XLS)
